# Resilience-by-Design and Resilience-by-Intervention in supply chains for remote and indigenous communities

**DOI:** 10.1038/s41467-022-28734-6

**Published:** 2022-03-02

**Authors:** Emerson Mahoney, Maureen Golan, Margaret Kurth, Benjamin D. Trump, Igor Linkov

**Affiliations:** 1 Wampanoag Tribe of Gay Head (Aquinnah), Noepe (Martha’s Vineyard), Vineyard Haven, MA USA; 2 US Army Engineer Research and Development Center, Concord, MA USA

**Keywords:** Risk factors, Government

## Abstract

Indigenous and remote communities face difficulties in times of supply chain disruption. Here the authors comment on challenges faced by the Tribal Population of Noepe (Martha’s Vineyard) and argue for the inclusion of Resilience-by-Design and Resilience-by-Intervention in supply chain management.

## Teaser

The COVID-19 pandemic has illustrated the fragility of food security and associated supply chains for remote and Indigenous communities. Here we highlight challenges faced by the Tribal Population of Noepe (Martha’s Vineyard) and argue for the inclusion of Resilience-by-Design and Resilience-by-Intervention in supply chain management.

The coronavirus pandemic has underscored the cascading consequences of unexpected disruptions to food supply chains as well as the complexity of the public and private interactions governing the global food system. The impacts of those disruptions, compounded by systemic threats and vulnerabilities, such as climate change, socioeconomic disparities, and lean supply chain networks, are wreaking havoc on equitable and predictable nutritious food distribution and access^1^. Marginalized and remote communities with fewer geographic and financial connections and resources are more likely to struggle to overcome effects of fragile systems and maintain food security. Supply chain disruptions, amplified by the coronavirus pandemic, have brought into sharp focus the food insecurity for these communities.

In this comment, we highlight food supply chain issues facing the Tribal community on Martha’s Vineyard (MV). There is an urgent need for a deeper understanding of the repercussions of efficiency-driven supply chain operations for remote Tribal populations in the United States, which examines the importance of approaching food supply chain design and implementation from a resilience-seeking perspective to ensure equitable access to food. We contend that food security can be bolstered with a more nuanced supply chain configuration that targets resilience to cope with disruptions but does not compromise efficient movement of goods under normal operations^2,3^. Specifically, we suggest that Resilience-by-Design (RbD) and Resilience-by-Intervention (RbI) can be pursued as concurrent strategies to enhance the resilience of communities and their supply chains—to absorb stress and shocks to minimize disruptions, recover functionality if it is lost, and enhance adaptation for the future^4^.

## Food security on Martha’s Vineyard

Approximately 4.5 miles off the mainland coast of Massachusetts on the small island of Noepe (Martha’s Vineyard), the 1000 on-island members of the Wampanoag Tribe of Gay Head Aquinnah rely on a single ferry service to deliver most bulk food items to their stores. The coronavirus pandemic has disrupted ferry reliability and subsequent food availability at grocery stores and food pantries, however pandemic-related disruptions are compounded by pre-existing and systemic socioeconomic factors. Tribal Members have long had no alternatives to expensive and vulnerable Island supermarkets, as the growing seasonal influx of tourists and year-round island population, and increasingly expensive ferry transportation prices have made it nearly impossible for Tribal Members to travel off the Island without significant planning and financial burden.

Further, increasing climate change-related stressors are also compounding food security problems. Decreased ferry reliability due to turbulent weather conditions has made the food supply less reliable and more expensive. Harmful algal blooms are affecting the contribution of shellfish harvesting to the food supply, decreasing self-sufficiency in terms of both sustenance and economy. Such climatic factors have contributed to the current state of food insecurity facing many in the Tribe^5^. Beyond the impact of the coronavirus pandemic and the effects of a changing climate, emerging threats to food supply chain components, such as cyber-attacks, are also of growing concern, as evidenced by the June 2021 ransomware attack on the island’s ferry service^6^. Additionally, the federal government has failed to provide timely economic aid to the Aquinnah Wampanoag Tribal population, further limiting the Tribe’s purchasing power surrounding the transportation of bulk food items^5^.

Food supply chain failure is not unique to the Wampanoag Tribe. Where detailed statistics exist, rates of food insecurity as high as 92% have been noted for Tribes in the Klamath River Basin^7^. Further afield, the Inuit in Canada are also facing food insecurity due to fragile supply chains that provide food to their remote communities and the compounded impacts of environmental degradation from climate change and globalization^8^. A number of Amazonian Tribes in Peru, such as the Shawi, have cited reduced hunting and fishing access in addition to environmental degradation as causes of disrupted food supply chains and high rates of food insecurity^9^. While case studies have been conducted on the aforementioned indigenous groups, we lack a clear understanding of food security issues facing the majority of Tribes. In large surveys, such as that from the U.S. Department of Agriculture (USDA), figures for Native Americans are grouped together in an “other” category^10^. This grouping fails to adequately capture the variation in the drivers of food security issues unique to Native American communities.

## Issues with traditional supply chain and risk management approaches

The necessity for resilience thinking in food supply chains is a product of existing overemphasis on creating lean and efficient networks, which have traditionally sought to limit overhead costs such as inventory, storage, and redundant transportation routes^11^. Under normal operations, efficiency-driven networks aim for maximum return-on-investment and longevity of limited resources at a low consumer cost^12^. The design of efficient supply chains is based on traditional risk management approaches that identify and mitigate against historical threats, with the goal of avoiding costs from disruptive events.

However, traditional strategies cannot be relied upon for outlier or compounding events, such as the coronavirus pandemic^13^. For example, pandemic-related disruptions to agricultural production in the U.S. created a supply-side shock from the reduced output caused by social distancing in meat packing plants, as well as a demand-side shock from the change in packaging and distribution channels required to meet changing consumer habits, which propagated supply chain failure throughout the country^14^. The impact of the pandemic on shipping logistics, unemployment, trade politics, quarantine policies, and inflation have further compounded disruptions within the highly interconnected global food network^15,16^. Agriculture and food markets are typically reliant on efficient, just-in-time manufacturing and delivery, and struggle to adapt and recover in the face of systemic shocks^15,17^. Demand-side shocks such as consumers’ loss of income and the availability of safety nets cannot be understated, as the end-user is forced to cope with the inability of the food supply chain to recover^18^.

Martha’s Vineyard is not immune to systemic adaptation and recovery issues in traditional supply chains, with weak points in island food supply exposed during the pandemic. From the Tribe’s perspective, there have been shortages and lags in government funding, while access to normal channels of local aid have diminished and Tribal Members have been hard-hit by unemployment. Local supermarket owner, Steve Bernier, reported that ten months post-pandemic, his supermarket had only received about 30% of the products in each of his orders. Bernier attributed this to a failure to adapt to a system only designed for efficiency and stakeholder profits^19^.

**Fig. 1:**
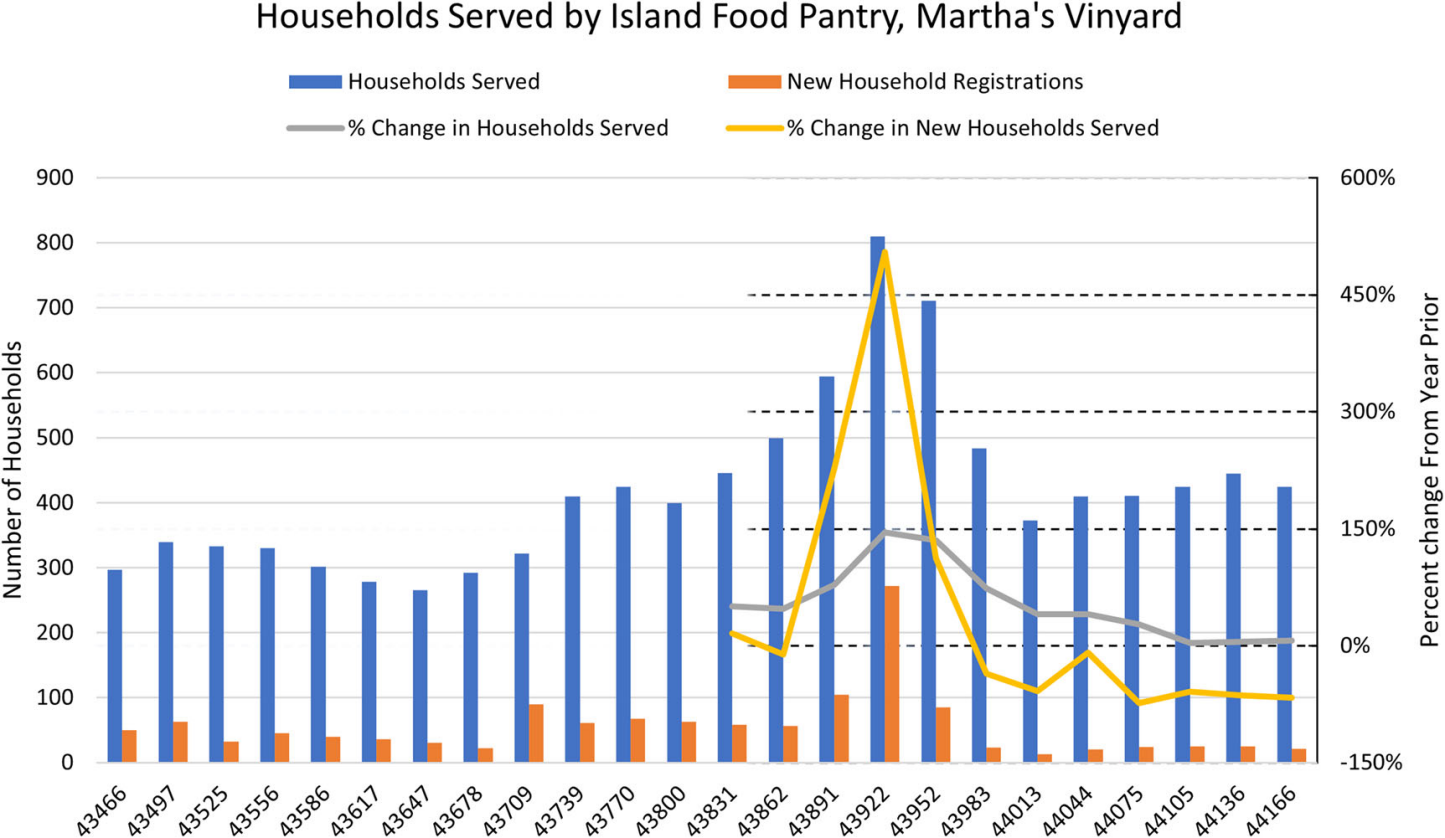
Monthly count of households served by the largest food bank on Martha’s Vineyard (data from ref. ^20^). First case of COVID-19 confirmed in the Boston area February 1, 2020 and a state of emergency issued by the governor on March 10, 2020, spurring a spike in the number of households seeking food assistance on Martha’s Vineyard. This shift in the food supply chain required outside resources be delivered to the Island Food Pantry in order to meet critical demand.

While there have been local efforts on Martha’s Vineyard to cope with food insecurity during the coronavirus pandemic, these have been hampered by infrastructural problems. The Island Grown Initiative—a grassroots organization dedicated to local food production and distribution, equity, and education—increased agricultural production but struggled to deliver food because they did not have the infrastructure to expand their logistics^20^. Similarly, the Island Food Pantry experienced a drastic increase in demand for food during the initial coronavirus pandemic outbreak (see Fig. 1), yet their small and rigid infrastructure was unable to withstand the increase. According to one interviewee, this lead to hundreds of pounds of lost food before state funding allowed for organizational expansion^21^.

Food supply and demand issues during the coronavirus pandemic are not unique to Martha’s Vineyard, with food banks across the United States struggling to meet demand. The non-profit Feeding America approximates that one in six households across the U.S. have experienced difficulty securing food, causing food bank and philanthropic aid demand to increase 60% nationally^22^. By contrast, in April 2020 MV experienced a 450% increase in households seeking assistance. Food insecurity is known to be related to factors of poverty and access^23^. The experience of MV highlights the role of access as a driver of food insecurity; physical remoteness and food supply shortages at points in time during the pandemic have contributed to the food security status of local communities. Understanding supply chain design and food insecurity trends is a first step in facilitating the development of a portfolio of corrective actions, guided by the new resilience-based perspectives described below, and complementary to risk management.

## Resilience-by-Design and Resilience-by-Intervention: two ways to confer capacity for system recovery and adaptation to disruption

Resilience, as a system objective, is concerned with maintaining the intended function of a system during normal times as well as under stress and shocks. A food system’s function of providing food to households and individual consumers can persist with the help of different components and capabilities that are permanent fixtures or that take effect when they are needed. A portfolio to enhance the resilience of societal functions can include strategies targeting physical infrastructure, policies, mutual agreements, and communication channels, and can reside internal or external to the system that provides the functions of interest. We propose Resilience-by-Design (RbD) and Resilience-by-Intervention (RbI) as a useful framing to help conceptualize the wide range of potential corrective actions that may comprise a comprehensive and cost-effective resilience strategy^24,25^.

A system possessing RbD can maintain and recover its critical functions autonomously. As an approach, RbD identifies elements that can be implemented within the system to minimize the degradation of critical functions under stressors and shocks, and facilitate rapid recovery if function is lost. In food supply chains, production and transportation networks should be organized in such a way as to ensure self-sufficiency, which may aid in overcoming negative consequences of disruptions. RbI, by contrast, utilizes resources that are external to the system to facilitate the persistence and recovery of critical functions. RbI strategies can take effect when the system cannot sustain critical functions on its own; they can act as emergency stop-gap measures to assist with the delivery of critical functions and allow the system to recover and/or actively support recovery.

In food supply chains, RbD strategies may include the involvement of multiple logistics networks or retailers that are capable of performing the same function (i.e., adding redundancy into the supply chain), back-up suppliers, adaptable feedstock capabilities, local food production, or re-allocation of food. RBI strategies may include establishing emergency government subsidies to producers (e.g., farm subsidies) or consumers, (e.g., Supplemental Nutrition Assistance Program), government stockpiles, or mandatory nutritional guidelines for school lunch programs. Ideally, RbI strategies are conceived and arranged prior to a disruption so they can take effect rapidly. Developing a portfolio of appropriate RbD and RbI corrective actions requires resilience analytics to strategize implementation and critically consider tradeoffs, such as where efficiencies can be sacrificed for the sake of enhancing resilience. Having both RbD and RbI in the supply chain management toolbox facilitates the balance of governance, resources, and priorities for all stakeholders. In so doing, RbD and RbI help to frame a systems approach to ensuring supply chains maintain optimal performance (e.g., food security) despite disruptions.

On Martha’s Vineyard, the Vineyard Food Equity Network has employed RbD strategies, and has been a key player in affording some internal capacity for resilience during the pandemic. For example, the lead member of the network, Island Grown Initiative, doubled their local agricultural production from the beginning of the pandemic and saved close to 23 tons of food from spoilage at MV supermarkets and commercial farms^20^. Despite initial challenges with expanding distribution, their efforts effectively diversified the fresh produce supply chain, minimized reliance on the off-Island transportation links, and restructured the waste management portion of the supply chain. This highly local experience exemplifies that the use of different strategies to enhance resilience come with different challenges; the Island Grown Initiative was able to quickly adapt its production capacity, but the connections necessary for distribution required more coordination and resources, limiting speed of response.

In terms of RbI, a primary strategy on MV during the pandemic has been in the form of state infrastructure grants made possible by the Food Security Infrastructure Grant Program. Island Food Pantry used the funding to buy a refrigerated van to enable a new food delivery service^21^. Such ad hoc measures have been essential on MV during the pandemic and indicate the need for resilience planning that seeks an optimal combination of RbD and RbI measures to prevent food insecurity under future scenarios and avoid the costs that accompany purely ad hoc measures.

While the island developed many measures to ensure food security for its general population and the Tribal community since the onset of the pandemic, pre-existing weak points exposed in its food system could be improved upon in the future with RbD and RbI measures. For instance, shelves were often bare at the Stop & Shop grocery stores, and due to staff shortages and COVID outbreaks, one of the two Cronig’s Market locations was forced to shut down^19^. This further concentrated the remaining grocery stores on the east side of the island, increasing the travel burden for west island Tribal community members to access food. Additionally, typical government RbI measures like food subsidies are difficult for many non-English speaking, food insecure island residents to navigate, and act as a significant deterrent to the exact population these programs target^21^. Incorporating additional outreach (RbI) and internal community networks (RbD) could help to enhance this ineffective RbI measure. Other island-based RbD efforts can come from local food production, a movement that has been increasingly propelled by the Food Equity Network. Involving a variety of different farms, businesses, and families to develop a transparent local food production system will allow residents to rely less on imports from the fragile supply chain to the island.

## Resilience-by-Design and Intervention for complex systems: future outlook

A step towards reducing food security should begin with the implementation of Resilience-by-Design and Resilience-by-Intervention within more traditional efficiency-driven and risk management supply chain management. Table 1 provides a general framework for these approaches to be developed in the context of food supply chains. The basis of the framework is built upon the National Academy of Science definition of resilience, that disaggregates the disaster event cycle into four stages: preparation, absorption, recovery, and adaptation^26^. The benefits of resilience analytics extend to being more prepared for various types of disasters, creating systematic, tiered approaches to combating threats to supply chains, and incorporating a broad network view with perspectives of stakeholder and societal goals. Better understanding how internal and external stakeholders and networks interact, governments and non-governmental organizations can ensure they best serve public interest through fully leveraging the portfolio of strategies available to them and private food producers and distributors.

**Table 1:** Key differences between traditional supply chain (SC) risk management, Resilience-by-Design and Resilience-by-Intervention.

	Traditional supply chain management approaches	Resilience-by-design	Resilience-by-intervention
Threats to food security/supply chains	Systemic (climate change, social and economic changes) and shocks (pandemics, cyber-attacks, natural disasters)		
Actions and analytics/stages	Hardening the system based on assessing largely known or predictable risks (i.e., product of threat, vulnerability, and consequence) for preparation and absorption of threats.	Engineering systems to be recoverable and adaptable in response to both predicted and unknown threats based on modeling loss of critical system functionality over time.	Resources outside an individual SC (e.g., stockpiles, services, community stakeholder, etc.) available to facilitate recovery and adaptation of systems in case of disruptions based on modeling loss of critical system functionality over time.
Advantages of approach	Methodology is well developed and practiced, allowing the system to retain functionality without disruptions. Works well for known or predictable threats.	System is designed for self-healing and is able to quickly respond to either known/predictable or unknown disruptions in the context of its own needs and abilities. Can also make the system more agile.	Combined resources and capabilities allow for cost saving as well as flexibility to adapt to a much broader range of possible disruptions.
Disadvantages of approach	Approach limited to known or predictable threats; cost increases exponentially once low-probability high consequence disruptions are considered. Possible catastrophic failure since systems are not designed for recovery.	System needs to maintain redundant capabilities and training of personnel to continue to function and act accordingly. May be quite expensive.	Necessary cooperation and resource allocation among stakeholders, regulators, and other SC players limits speed/viability of corrective action development. Cost may be substantial, but lower than in by-design.

Failures of efficiency-driven supply chains are not unique to MV, the Klamath River basin region, the Inuit, or to the coronavirus pandemic. Remote populations continue to face numerous challenges related to the supply chains underpinning their food security, from high reliance on imported food to exposure to climate stressors^27,28^. Leveraging resilience analytics can harmonize the multiple objectives driving food supply chain operation and implementation. System weaknesses can be identified through stress testing network configurations, while corrective actions can be evaluated by how well they prevent overall losses in system function over time. Proactive RbD and RbI strategies for maintaining food security in light of supply chain disruption show promise for remote and marginalized communities, highlighted here via the example of the Aquinnah Wampanoag Tribe on Martha’s Vineyard. At the highest level, government regulation needs to ensure transparency for large corporations’ food supply chains, devote more resources to local food production and emergency food supply strategies to marginalized communities, and subsidize large scale food distribution programs. Changing the system entirely, however, requires efforts on behalf of community business owners and municipal governments to devise strategies to combat food supply threats as well. Intentionally leveraging corrective action from a RbD and RbI perspective allows for both internal and external agency over the food supply chain, which may increase food security and equity.
